# Genome-Wide Analysis of the *Sucrose Synthase* Gene Family in Grape (*Vitis vinifera*): Structure, Evolution, and Expression Profiles

**DOI:** 10.3390/genes8040111

**Published:** 2017-03-28

**Authors:** Xudong Zhu, Mengqi Wang, Xiaopeng Li, Songtao Jiu, Chen Wang, Jinggui Fang

**Affiliations:** Nanjing Agricultural University, Weigang 1 hao, 210095 Nanjing, China; 2014204002@njau.edu.cn (X.Z.); 2014104015@njau.edu.cn (M.W.); 2014204003@njau.edu.cn (X.L.); 2015204003@njau.edu.cn (S.J.); wangchen@njau.edu.cn (C.W.)

**Keywords:** grapevine, sucrose synthase, syntenic analysis, phylogenetic tree, expression profile

## Abstract

Sucrose synthase (SS) is widely considered as the key enzyme involved in the plant sugar metabolism that is critical to plant growth and development, especially quality of the fruit. The members of *SS* gene family have been identified and characterized in multiple plant genomes. However, detailed information about this gene family is lacking in grapevine (*Vitis vinifera* L.). In this study, we performed a systematic analysis of the grape (*V. vinifera*) genome and reported that there are five *SS* genes (*VvSS1–5*) in the grape genome. Comparison of the structures of grape *SS* genes showed high structural conservation of grape *SS* genes, resulting from the selection pressures during the evolutionary process. The segmental duplication of grape *SS* genes contributed to this gene family expansion. The syntenic analyses between grape and soybean (*Glycine max*) demonstrated that these genes located in corresponding syntenic blocks arose before the divergence of grape and soybean. Phylogenetic analysis revealed distinct evolutionary paths for the grape *SS* genes. *VvSS1/VvSS5*, *VvSS2/VvSS3* and *VvSS4* originated from three ancient *SS* genes, which were generated by duplication events before the split of monocots and eudicots. Bioinformatics analysis of publicly available microarray data, which was validated by quantitative real-time reverse transcription PCR (qRT-PCR), revealed distinct temporal and spatial expression patterns of *VvSS* genes in various tissues, organs and developmental stages, as well as in response to biotic and abiotic stresses. Taken together, our results will be beneficial for further investigations into the functions of *SS* gene in the processes of grape resistance to environmental stresses.

## 1. Introduction

Sucrose is an essential element of life cycle in higher plants. It is mainly produced by photosynthesis in source tissues and is exported to sink tissues where it serves as a carbon source of energy for various metabolic pathways [1,2]. In addition, when suffering from environmental stress such as cold, drought and salt stress, the accumulation of sucrose protects the stability of both membranes and proteins in plant cells. Further, sucrose is reported to supply energy to increase metabolism when plants recover from these stresses [3,4]. In recent years, it has also been recognized that sucrose can act as important signal in plants to modulate a wide range of processes through regulating the expression level of genes encoding enzymes, storage and transporter proteins [5,6,7,8]. Moreover, sucrose also participates in several fundamental processes, such as cell division [9], flowering induction [10], vascular tissue differentiation [11], seed germination [12], and the accumulation of storage products [13]. Thus, the study of the sucrose metabolism is pivotal in understanding various sides of plant physiology.

Sucrose synthase (SS) is one of the key enzymes that regulate the sucrose metabolism in plant. SS catalyzes the reversible reaction of sucrose and uridine diphosphate (UDP) into uridine diphosphate glucose (UDP-glucose) and fructose [14,15]. SS enzymes have pivotal roles in a large range of plant metabolic processes, such as sucrose distribution between plant source and sink tissues [16,17,18,19], starch biosynthesis, cellulose synthesis in secondary cell wall [20], nitrogen fixation [21] and response to abiotic stresses [14,18,22].

The identification and characterization of the sucrose synthase genes is the first step towards detecting their specific roles involved in different types of metabolic pathways. With the whole-genome sequencing data of many plants being released in such a short period, an increasing number of *SS* gene families have been identified subsequently and characterized. The number of members of the small multigene family differs among the plant species examined. For example, three *SS* genes were identified in the maize and pea (*Pisum sativum*) genomes [23,24]; there exist six distinct *SS* gene members in Arabidopsis, rice, *Hevea brasiliensis* and *Lotus japonicus* [22,25,26,27]; and diploid cotton (*Gossypium arboreum* and *Gossypium Raimondii*) genomes each contain eight *SS* genes [28]. Both of the tetraploid cotton (*G. Hirsutum*) [28] and poplar (*Populus trichocarpa*) [29] genome contain fifteen *SS* members, which belonged to one of the largest family of *SS* genes identified to date [30]. In all cases, the spatio-temporal expression patterns of the different *SS* genes across tissue types within each plant species implied that each member of *SS* gene families performed specific physiological functions in a given tissue/organ. For instance, the expression profiling of Arabidopsis *SS* genes reveals partially overlapping but distinct patterns. *AtSS2* is expressed highly only in a critical period of the seeds; in contrast, *AtSS1* and *AtSS5* are much more widely expressed, in the root, stem, flower, silliques and seed. *AtSS6* is expressed mainly in the root, flower and seed [31]. *PsSS1*, *PsSS2*, and *PsSS3* genes are expressed predominately in seed, leaf, and flower of the pea, respectively [23]. Tissue/developmental-specific expression patterns of *SS* genes have also been demonstrated in many other plant species, such as *L. japonicus*, rice, and citrus [26,27,32,33]. In conclusion, extensive studies about the *SS* gene family have been carried out in various plant species such as Arabidopsis, rice, citrus, and poplar, however, few studies about the *SS* genes in grape have been done till date.

In most fruit crops, fruit quality is determined by the contents of sugars (such as sucrose), and sucrose synthase activity can significantly influence the sucrose accumulation. Thus, the study of sucrose synthase is key to the issue of fruit quality improvement. Grapevine (*V. vinifera*) is one of the most important fruit crops in the world [34] and annually production exceeding 78.6 million tons in 2014 (FAOSTAT) [34]. The quality improvement of grape berry is a very important issue. However, prior work focused on the biological functions of SS isozymes, and there is little information on the comprehensive analysis of *SS* gene family in grapevine. Shangguan et al. [35] have identified five putative grape *SS* gene, however to date the characterization of structure, expression patterns and evolution history in the grape *SS* gene family remains elusive. To fully understand the molecular biology, evolution and possible functions of the grape *SS* gene family, we must characterize the member and their evolutionary relationships.

In the present study, we employed bioinformatics methods to analyze the characters of grape *SS* genes on a genome-wide scale in *V. vinifera* based on several publicly available data. Furthermore, we investigated their expression profiles of the grape *SS* genes at different tissues and in response to various stresses. These comprehensive results will provide an insight that will assist in better understanding the potential functions of SS enzymes in sucrose transport or sugar accumulation of grape plants in further studies.

## 2. Methods

### 2.1. Mining of Grape Sucrose Synthase Genes

To verify a complete list of grapevine *Sucrose Synthase* (*SS*) genes, we downloaded the annotated grapevine proteins from three public databases: the National Centre for Biotechnology Information (NCBI. Available online: http://www.ncbi.nlm.nih.gov/), the Genoscope (Grapevine Genome Browser. Available online: http://www.genoscope.cns.fr/externe/GenomeBrowser/Vitis/) and the Grape Genome Database (CRIBI. Available online: http://genomes.cribi.unipd.it/grape/, V2.1). Then, the Hidden Markov Model (HMM) profiles of the core sucrose synthase domain (PF00862) and Glycosyl transferases domain (PF00534) from Pfam database (Pfam. Available online: http://pfam.xfam.org/, 30.0) was downloaded and used to survey all grapevine proteins in the 12X coverage assembly of the *V. vinifera* PN40024 genome.

All putative *SS* genes were manually verified with the InterProScan program (InterProScan. Available online: http://www.ebi.ac.uk/Tools/pfa/iprscan5/) and the Conserved Domains Database (CDD. Available online: http://www.ncbi.nlm.nih.gov/cdd) to confirm their completeness and existence of the core domains. Length of sequences, molecular weights and isoelectric points of deduced polypeptides were calculated by using tools provided at the ExPasy website (ProtParam. Available online: http://web.expasy.org/protparam/). Finally, manual annotation was performed to resolve any discrepancy between incorrectly predicted genes and the actual chromosomal locations of involved genes in question. In addition, we also use “sucrose synthase” as a query keyword to search the grapevine gene at NCBI Gene database (NCBI. Available online: https://www.ncbi.nlm.nih.gov/gene/). As *sucrose phosphate synthase* genes also possess these two domains except for sucrose-phosphatase domain (PF05116) in N-terminal, the candidate sequences of grape *SS* genes obtained from these three genome databases were submitted to InterPro and CDD databases to confirm the existence of these two domains (PF00862 and PF00534) and nonexistence of the sucrose-phosphatase domain. Those protein sequences lacking the sucrose synthase domain and Glycosyl transferases domain were removed and the longest variant with alternative splice variants were selected for further analysis.

The *SS* sequences of plant species, such as Arabidopsis, rice, maize, and poplar, were collected by searching the NCBI database (NCBI. Available online: https://www.ncbi.nlm.nih.gov), the Arabidopsis Information Resource (TAIR. Available online: http://www.arabidopsis.org/, Phytozome (Phytozome. Available online: https://phytozome.jgi.doe.gov/pz/portal.html, V11), rice genome database in the rice genome annotation project (TIGR. Available online: http://rice.tigr.org), and maize genome database (MaizeGDB. Available online: http://www.maizegdb.org/), using “sucrose synthase” as a query keyword. Other plant species such as *Solanum tuberosum* [14], *Lycopersicon esculentum* [30], *Triticum aestivum* [30], *Hordeum vulgare* [30], derived from some related articles.

### 2.2. Chromosomal Localization and Syntenic Analysis

Grape *SS* genes were mapped to chromosomes by identifying their chromosomal locations based on information available at the Grape Genome Database (CRIBI. Available online: http://genomes.cribi.unipd.it/grape/, V2.1). The segmental and tandem duplication regions, as well as chromosomal location, were established using PLAZA (PLAZA. Available online: http://bioinformatics.psb.ugent.be/plaza/versions/plaza_v3_dicots/, v3.0 Dicots). For syntenic analysis, synteny blocks within the grape genome and between grape and soybean genomes were downloaded from the Plant Genome Duplication Database and visualized using Circos (Circos. Available online: http://circos.ca/).

### 2.3. Gene Structure and Phylogenetic Analysis

The intron-exon organization analysis was carried out using Gene Structure Display Server (GSDS. Available online: http://gsds.cbi.pku.edu.cn/, 2.0) by alignment of the complementary DNA (cDNA) sequences with their corresponding genomic DNA sequences. Multiple alignments of the identified grape SS amino acid sequences were performed using ClustalX [36]. The phylogenetic tree was constructed with MEGA5.1 using the Maximum Likelihood and the bootstrap test carried out with 1000 replicates [37].

### 2.4. Ratio of Nonsynonymous to Synonymous Substitutions (dN/dS) and Relative Evolutionary Rate Test

The coding sequences of the grape *SS* genes were aligned following the amino acid alignment by Codon Alignment (Codon Alignment. Available online: https://www.hiv.lanl.gov/content/sequence/CodonAlign/codonalign.html, v2.1.0). The numbers of nonsynonymous nucleotide substitutions per nonsynonymous site (dN) and the numbers of synonymous nucleotide substitutions per synonymous site (dS) were estimated with the yn00 program of PAML4 [38]. Tajima relative rate tests [39] were performed with amino acid sequences for the two grape *SS* duplicate pairs using MEGA5.1 [37].

### 2.5. Microarray Data Analysis

Microarray gene expression profiles of the different organs of grapevine at various developmental stages were downloaded from Gene Expression Omnibus (GEO, available online: https://www.ncbi.nlm.nih.gov/geo/) under the series entry GSE36128. The expression data obtained from the *V. vinifera* cv. “Corvina” (clone 48) expression atlas were normalized based on the mean expression value of each gene in all tissues/organs analyzed.

Expression analyses in response to abiotic and biotic stresses were based on microarray data (series matrix accession numbers GSE31594, GSE31677, GSE6404, GSE12842 and GSE31660) downloaded from the NCBI GEO datasets (GEO. Available online: https://www.ncbi.nlm.nih.gov/geo/). All the expression data were calculated as log2 fold change in treated vs. untreated samples.

The heat maps for genes expression level were created by HemI (The Cuckoo Workgroup, Wuhan, China).

### 2.6. Plant Material and Treatments

Two-year-old *V. vinifera* cv. Chardonnay seedlings were planted in plastic pots containing peat, vermiculite, and perlite (3:1:1, *v*/*v*) were grown under the following conditions: 28 °C day 12 h/18 °C night 12 h, and a relative humidity ranging from 70% to 85%, at the greenhouse of the Nanjing Agriculture University (Nanjing, China). When shoots of vines were 40–50 cm in length, the third and fourth fully expanded young grapevine leaves beneath the apex were selected for treatments and control. At each time point of each treatment, six leaves from six separate plants were combined to form one sample, and all the experiments were performed in triplicate.

For salt stress treatments, two-year-old soil-grown plants were irrigated with 100 mM NaCl. Treated leaves were collected at 0, 48, and 96 h post-treatment. Plants were sprayed with sterile water for control. For cold treatment, plants were first grown at 23 °C, and then transferred to 4 °C, and treated leaves were collected at 0, 24, and 48 h post-treatment. For high temperature treatment, plants were first grown at 23 °C, and then transferred to 42 °C, and treated leaves were collected at 0, 24, and 48 h post-treatment. Plants were grown at 23 °C for control. For drought treatment, the plants in plastic pots were first kept well-watered and then water was withheld to impose a water stress. Leaf samples were harvested at 0, 10 and 20 days after initiation of the treatments. Control plants were maintained in well-watered conditions. For dark treatment, the plants were first grown at the greenhouse, and then transferred to dark environment, and treated leaves were collected at 0, 48, and 96 h post-treatment. Plants were grown in the normal conditions for control.

These various tissues/organs such as leaves, flowers and berries of grapevine at different developmental stages were derived from six-year old *V. vinifera* cv. Chardonnay grapevine grown under normal cultivation conditions under rain shelter cultivation in Jiangpu Fruit Experimental Farms of Nanjing Agricultural University, Nanjing, China.

Tissue samples were immediately frozen in liquid nitrogen and stored at −70 °C until they were subjected to RNA extraction.

### 2.7. RNA Extraction, cDNA Synthesis and Quantitative Real-Time PCR (qRT-PCR)

Total RNA was extracted from each organ/tissue by using EZNA Plant RNA Kit (R6827-01, Omega Biotek, Norcross, USA) according to the manufacturer’s instructions. First-strand cDNA was synthesized from 1.0 μg total RNA by using M-MLV reverse transcriptase (Fermentas, Canada) according to the manufacturer’s instructions. Quantitative PCR (qPCR) was carried out using TransStart Tip Green qPCR SuperMix (TransGen Biotech, Beijing, China) on an IQ5 real time PCR machine (Bio-Rad, Hercules, CA, USA) according to the manufacturer’s instructions. The PCR was conducted by following procedure: predenaturation at 94 °C for 30 s, followed by 40 cycles of denaturation at 94 °C for 5 s, primer annealing at 60 °C for 15 s, and extension at 72 °C for 10 s. Optical data were acquired after the extension step, and the PCR reactions were subjected to a melting curve analysis beginning from 65 °C to 95 °C at 0.1 °C s^−1^. The grape *UBI* gene (Gene code LOC100259511) was used as an internal control. All reactions were performed in triplicate in each experiment and three biological repeats were conducted. Primers used for qRT-PCR are listed in Supplementary Table S3. Each relative expression level was analyzed with IQ5 software using the Normalized Expression method (2^−△△CT^ and 2^−△CT^ method). Expressional data consist of three replicated treatments and controls, which were calculated as 2-log-based values and were divided by the control.

## 3. Results

### 3.1. Characteristics of the Grapevine Sucrose Synthase Gene Family

We finally identified five non-redundant *sucrose synthase* genes in the three grapevine genome database, the five *SS* genes (*VIT_204s0079g00230*, *VIT_205s0077g01930*, *VIT_207s0005g00750*, *VIT_211s0016g00470* and *VIT_217s0053g00700*) were found in the V2.1 grape genome database hosted at CRIBI, and the corresponding five *SS* genes (LOC100266759, LOC100243135, LOC100267606, LOC100249279 and LOC100252799) were found in the NCBI. Similarly, five *SS* genes (*GSVIVT01035210001*, *GSVIVT01035106001*, *GSVIVT01028043001*, *GSVIVT01015018001* and *GSVIVT01029388001*) were found in the 12X grapevine genome database. The results of current analysis were consistent with previous studies [35], and no more members were identified. However, some minor difference in the properties of annotated *sucrose synthase* genes, such as the length of DNA and amino acid sequences, were noted between CRIBI V2.1 genome database and two other genome databases. Therefore, we selected the latest V2.1 grape genome database hosted at CRIBI released in 16 April 2015. Currently this V2.1 grapevine genome database is the latest one with 2258 new coding genes and 3336 putative long non-coding RNAs. Moreover, several gene models were improved and alternative splicing was described for about 30% of the genes. Based on these five genes’ distributions and relative linear orders on the respective chromosomes, we named them *VvSS1* to *VvSS5* according to their chromosomal locations. Characteristics of the five *SS* genes are shown in Table 1 and included the deduced protein length, the molecular weight, the isoelectric point, the aliphatic index and the grand average of hydropathicity. The size of these five *SS* genes varied from 4229 to 9214 bp, but the coding DNA sequence (CDS) sizes were all quite similar, around 2436 to 2610 bp. Molecular analysis of the full-length deduced polypeptides indicated that the putative proteins of these grape *SS* genes contain 811 to 906 amino acids (predicted 92.4 to 102.7 kDa in molecular weight) with their isoelectric point calculated ranging from 5.73 to 8.28. This range of variability implies that different *VvSS* proteins might operate in different microenvironments.

**Figure 1:**
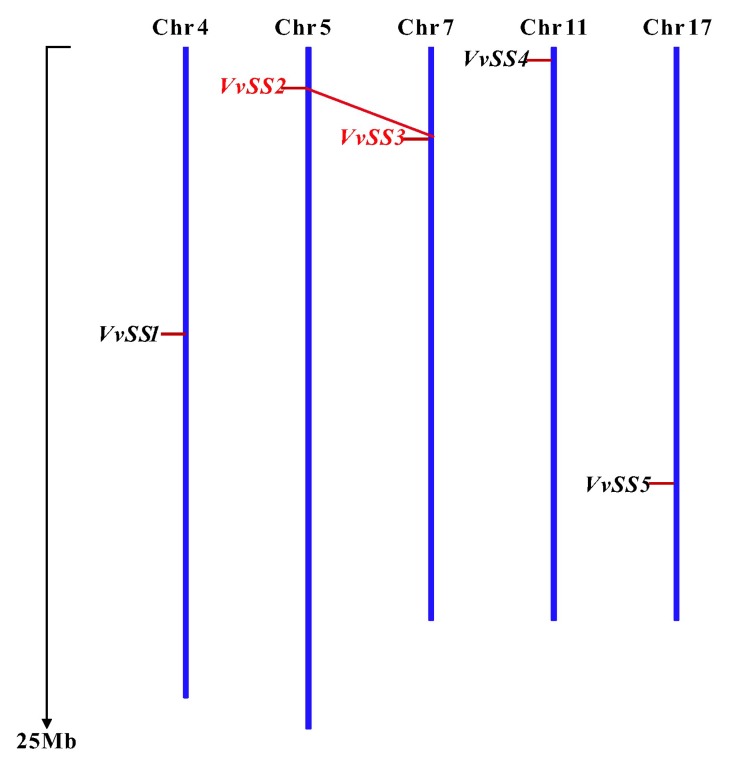
Chromosomal distribution of *Sucrose Synthase* (*SS*) genes in the grape genome. The chromosome number is shown at the top of each chromosome. The positions of the grape *SS* genes are marked by red lines on the chromosomes. The block duplication of *VvSS2–VvSS3* are linked by red lines.

As shown in Figure 1, five *SS* genes in grape were located on five chromosomes, and each chromosome contained only one gene. In addition, we also found *VvSS2* and *VvSS3* were located in duplicate block by the search in PLAZA v3.0 Dicots database (Figure 1). Sequence comparison revealed that the genes share a high sequence homology at the nucleotide level (54.85% to 77.55% identity) within the coding region and at the amino acid level (47.70% to 79.12% identity) (Table 2). Two unique *VvSS* pairs (*VvSS1–VvSS5* and *VvSS2–VvSS3*) were found, because a higher sequence identity of nucleotide and amino acids was observed between *VvSS*2 and *VvSS3*, *VvSS*1 and *VvSS5*. *VvSS4* gene was very different from the other five *VvSS* genes.

### 3.2. Exon/intron Organization of the Grapevine Sucrose Synthase Gene Family

**Figure 2:**
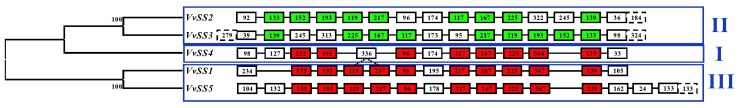
Phylogenetic relationships and intron-exon organization of grape *SS* genes. The unrooted phylogenetic tree was constructed using the full-length protein sequences of five grape *SS* genes by the Maximum Likelihood method with 1000 bootstrap replicates. The three groups are marked by square boxes and numbered with Roman numerals. The 5’ and 3’ untranslated regions (UTRs) are represented by a dashed box. Numbers in boxes represent the sizes (bp) of corresponding exons or UTR regions. The green and red boxes represent exons shared in Group I and II, respectively. The dashed line indicated the relationship between three exons. The solid lines represent introns whose size is not indicated.

Analysis of the exons/introns structures of gene can provide important insights into the molecular mechanism of evolution of gene family. To investigate the exon/intron structure of the grape *SS* genes, the CDS and genomic sequences were compared. As shown in Figure 2, these genes typically consist of 13, 15, and 16 exons interrupted by 12, 14, and 15 introns of varying sizes, respectively. These exons, with lengths of 96, 174, 117, 167 and 225, appear almost at the same positions in the CDS region of most grape *SS* genes, consistent with the exons in cotton and Arabidopsis [28]. Based on the length and position of the exons, three intron/exon structure models of the *VvSS* genes were revealed and designated as I, II and III, respectively. A comparison of the intron/exon structures in the coding regions revealed significant differences among *VvSS* models (Figure 2). For each *SS* group, the gene structure also show unique features: (a) Group III members (*VvSS1* and *VvSS5*) shared the identical exon/intron pattern, such as exons block 1 (with lengths 155, 193, 119, 217 and 96) and exons block 2 (with lengths 117, 167, 225, 567 and 139) arranged in the coding region in order. (b) In Group I (*VvSS4*), the fifth position was an exon with length 336 in *VvSS4*, and in *VvSS1* of Group III the fourth and fifth exons were 119 and 217 in length. Thus, we assumed that exon with length 336 in *VvSS4* was split into two exons (exon with length 119 and length 217, respectively) in *VvSS1* through the evolutionary history. (c) In Group II (*VvSS2* and *VvSS3*), if we ignored the minor differences in length of some exons (such as the first exon with length 92 in *VvSS2* and the last exon with length 98 in *VvSS3*, the last exon with length 36 in *VvSS2* and the first exon with length 39 in *VvSS3*, etc.), and considered the arrangement order of these exons, it was a reasonable hypothesis that *VvSS2* and *VvSS3* were duplicated and retained from whole-genome duplication event (WGD).

### 3.3. Gene Duplication and Syntenic Analysis of the Grapevine Sucrose Synthase Genes

**Figure 3:**
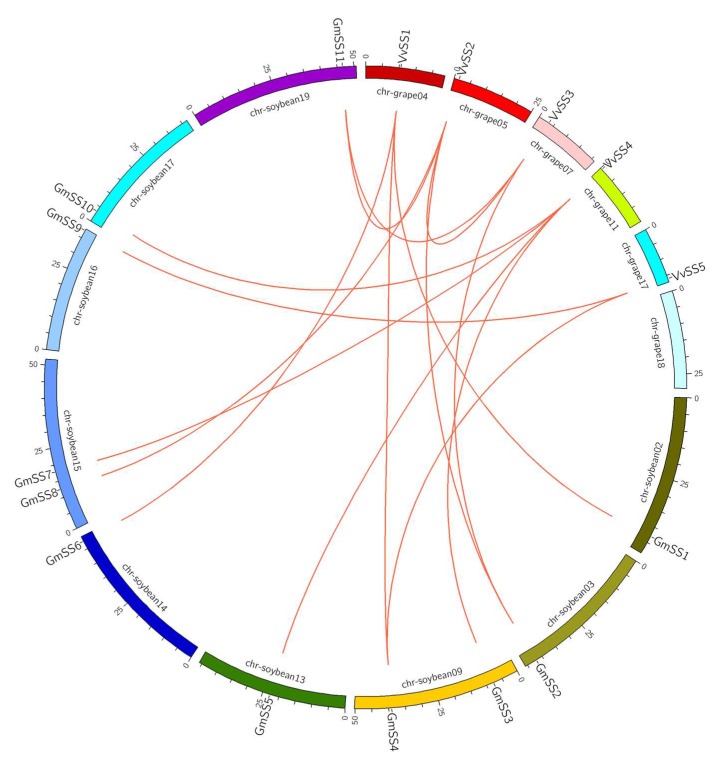
Segmental duplication of grape *SS* genes and syntenic analysis of grape and soybean *SS* genes. Chromosomes of *Vitis vinifera* and *Glycine max* are shown in different colors and in circular form. The approximate positions of the *GmSS* and *VvSS* genes are marked with a short black line on the circle. Colored curves denote the syntenic relationships between grape and soybean *SS* genes.

Gene duplication and divergence are important in gene family expansion [40] and the evolution of novel functions [41]. Grapevine has undergone whole-genome duplications during its evolutionary history [42]. To examine the impact of duplications on the *SS* gene family, we obtained tandem duplication and segmental duplication gene pairs from Plant Genome Duplication Database (PGDD. Available online: http://chibba.agtec.uga.edu/duplication/) and visualized them using Circos software (Circos. Available online: http://circos.ca/). In this study, we identified one segmental duplication pairs of grape *SS* genes (*VvSS2* and *VvSS3*) (Figure 3, Figure S1) but no tandem duplication events were detected in the grape *SS* genes. Further to explore the origin and evolution dynamics of grape *SS* genes, we investigated the syntenic relationship between grapevine and other plant species, and found that there is the syntenic relationship between grapevine and soybean (*Glycine max*) based on the results obtained from PGDD database. The syntenic analysis showed that syntenic genes included: *VvSS1-*Glyma.02G240400 (*GmSS1*)/Glyma.09G167000 (*GmSS4*)/Glyma.14G209900 (*GmSS6*), *VvSS2*-Glyma.03G216300 (*GmSS2*)/Glyma.15G151000 (*GmSS8*)/Glyma.19G212800 (*GmSS11*), *VvSS3*-Glyma.03G216300 (*GmSS2*)/Glyma.19G212800 (*GmSS11*), *VvSS4-*Glyma.13G114000 (*GmSS5*)/Glyma.17G045800 (*GmSS10*)/Glyma.15G182600 (*GmSS7*)/Glyma.09G073600 (*GmSS3*), and *VvSS5-*Glyma.09G167000 (*GmSS4*)/Glyma.16G217200 (*GmSS9*) (Figure 3, Supplementary Table S1). These results provide insights that will help infer the probable functions of grape *SS* genes.

### 3.4. Phylogenetic Analysis of the Grapevine Sucrose Synthase Genes

**Figure 4:**
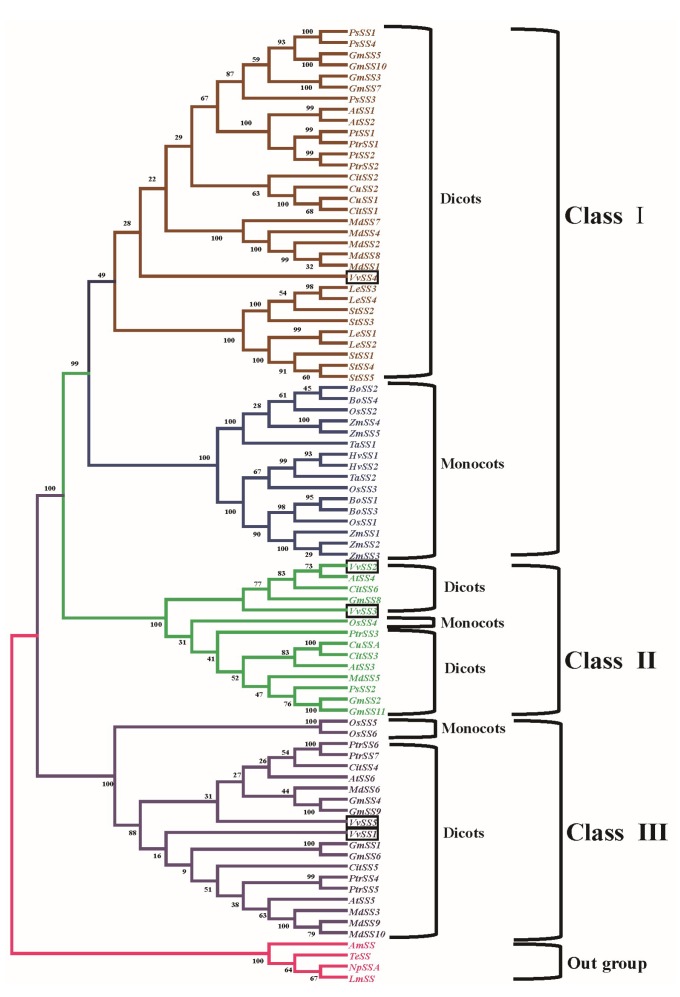
Phylogenetic relationships between the grape SS proteins and other SS homologous proteins in dicots and monocots (refer to Supplementary Table S2). *VvSS1* to *VvSS5* was highlighted in black boxes.

To investigate the evolutionary modules and their relationship to functional ones, we constructed a maximum likelihood tree using the full-length amino acid sequences of *SS* genes from 58 dicot sequences, 19 monocot sequences and four bacteria sequences based on 1000-replicate bootstrap values (Figure 4, Supplementary Table S2). The phylogenetic analysis revealed a relatively deep evolutionary origin and relatively recent duplications. All bacterial *SS* genes clustered into the same group, whereas those from the land plants formed a monophyletic group, showing that all plant *SS* genes originated from an ancestral type [43] (Figure 4). Bacteria *SS* genes were used as the outgroup; the plant *SS* genes can be categorized into three clearly distinct subgroups based on strong statistical support. These subgroups were designated as class I, class II and class III, respectively (Figure 4). The improved resolution in the tree also enabled us to make further subdivisions within classes I, II and III. Each class was resolved into two branches, one specific for dicots and another for monocots. Thus, the phylogenetic analysis suggested that most gene duplication events that gave rise to *SS* gene classes I–III occurred before the divergence of monocot/dicot. The five grape *SS* genes were distributed in the dicot branch of classes I, II and III, *VvSS4* in the dicot subgroup of Class I, *VvSS2* and *VvSS3* in Class II, and *VvSS1* and *VvSS5* in Class III, consistent with the exon/intron organization pattern and nucleotide/amino acid sequence identity (Table 2 and Figure 2). *VvSS4* itself was clustered into a branch; *VvSS2* was clustered together with *CitSS6*; *VvSS3* was closer to *OsSS4*; *VvSS5* was closer to *CitSS4* and *MdSS6*; and *VvSS1* was closer to *CitSS5*. The apparent diversification within the grape *SS* family could imply discrete biological roles for the paralogs despite their high sequence similarity.

### 3.5. Evolution of the Coding Sequences of the Grape SS Gene Pairs

Modes of selection can be estimated by the ratio of the numbers of nonsynonymous substitutions per nonsynonymous site (dN) to the numbers of synonymous substitutions per synonymous site (dS), that is, dN/dS > 1 indicates positive selection; dN/dS < 1, purifying selection; and dN/dS = 1, neural selection [44]. The combination of phylogenetic, exon/intron structure, and paragon analyses revealed two pairs of grape *SS* gene pairs (Table 2). The nonsynonymous substitution rates of two gene pairs are markedly higher than their synonymous substitution rates and their dN/dS values are >1 (Table 3), suggesting that these duplicates likely have been subject to positive selection. Furthermore, Tajima [39] relative rate tests were conducted to investigate whether one of the *SS* gene pairs has evolved at an accelerated rate following the duplication. A statistically significant increase in evolutionary rate occurs in *VvSS2/VvSS3* duplicated pairs (Table 4).

### 3.6. Expression Analysis of VvSS Genes in Different Organs, Tissues and Developmental Stages

To investigate the spatial and temporal expression patterns of *SS* genes in grapevine, we retrieved the data from a global transcriptomic atlas comprising 54 tissues, organs or developmental stages [45].

**Figure 5:**
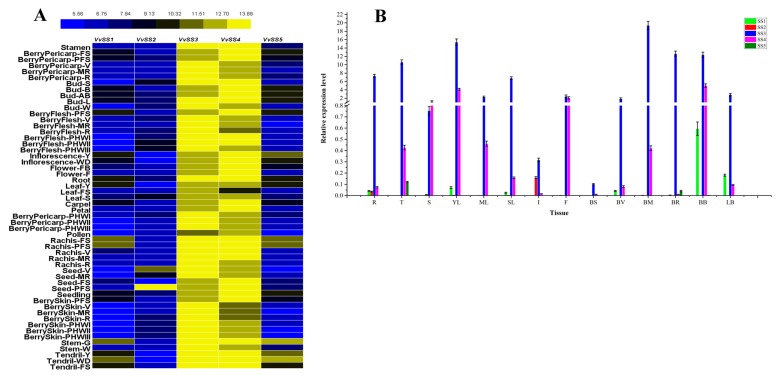
Expression profiles of the grapevine *VvSS* genes in different grapevine organs, tissues and developmental stages. (**A**) Expression of *VvSS* genes in the *V. vinifera* cv “Corvina” atlas (GEO Accession: GSE36128). Data were normalized based on the mean expression value of each gene in all tissues analyzed. The mean expression values were again normalized using logarithm with the base of 2 using the HemI software. Yellow and blue boxes indicate high and low expression levels, respectively, for each gene. **BerryPericarp-FS**: berry pericarp fruit set; **BerryPericarp-PFS**: berry pericarp post-fruit set; **BerryPericarp-V**: berry pericarp véraison; **BerryPericarp-MR**: berry pericarp mid-ripening; **BerryPericarp-R**: berry pericarp ripening; **Bud-S**: bud swell; **Bud-B**: bud burst (green tip); **Bud-AB**: bud after-burst (rosette of leaf tips visible); **Bud-L**: latent bud; **Bud-W**: winter bud; **BerryFlesh-PFS**: berry flesh post fruit set; **BerryFlesh-V**: berry flesh véraison; **BerryFlesh-MR**: berry flesh mid-ripening; **BerryFlesh-R**: berry flesh ripening; **BerryFlesh-PHWI**: berry flesh post-harvest withering I; **BerryFlesh-PHWII**: berry flesh post-harvest withering II; **BerryFlesh-PHWIII:** berry flesh post-harvest withering III; **Inflorescence-Y**: young inflorescence (single flower in compact groups); **Inflorescence-WD**: well developed inflorescence (single flower separated); **Flower-FB:** flowering begins (10% caps off); **Flower-F**: flowering (50% caps off); **Leaf-Y:** young leaf (pool of leaves from shoot of 5 leaves); **Leaf-FS:** mature leaf (pool of leaves from shoot at fruit set); **Leaf-S:** senescencing leaf (pool of leaves at the beginning of leaf fall); **BerryPericarp-PHWI:** berry pericarp post-harvest withering I (1st month); **BerryPericarp-PHWII**: berry pericarp post-harvest withering II (2nd month); **BerryPericarp-PHWIII:** berry pericarp post-harvest withering III (3rd month); **Rachis-FS:** rachis fruit set; **Rachis-PFS:** rachis post fruit set; **Rachis-V:** rachis véraison; **Rachis-MR:** rachis mid-ripening; **Rachis-R:** rachis ripening; **Seed-V:** seed véraison; **Seed-MR:** seed mid-ripening; **Seed-FS:** seed fruit set; **Seed-PFS:** seed post fruit set; **BerrySkin-PFS:** berry skin post fruit set; **BerrySkin-V:** berry skin véraison; **BerrySkin-MR:** berry skin mid-ripening; **BerrySkin-R:** berry skin ripening; **BerrySkin-PHWI:** berry skin post-harvest withering I; **BerrySkin-PHWII:** berry skin berry skin post-harvest withering II; **BerrySkin-PHWIII:** berry skin post-harvest withering III; **Stem-G:** green stem; **Stem-W:** woody stem; **Tendril-Y:** young tendril (pool of tendrils from shoot of 7 leaves); **Tendril-WD:** well developed tendril (pool of tendrils from shoot of 12 leaves); **Tendril-FS:** mature tendril (pool of tendrils at fruit set). (**B**) qRT-PCR validation of *VvSS* expression in different tissues obtained from two-year-old grapevines of *V. vinifera* cv. Chardonnay seedlings. Transcripts were normalized to the expression of the actin gene. The mean ± s.d. of three biological replicates are presented. **R**: principal root; **T**: tendrils from shoot of 7 leaves; **S**: green stem; **YL**: young leaf, leaves from shoot of 5 leaves; **ML**: mature leaf, leaves from shoot at fruit set; **SL**: senescencing leaf, leaves at the beginning of leaf fall; **I**: inflorescence, well developed inflorescence (single flower separated); **F**: flower, flowering (50% caps off); **BS**: berry post fruit set; **BV**: berry véraison; **BM**: berry mid-ripening; **BR**: berry ripening; **BB**: bud burst (green tip); **LB**: latent bud.

The expression patterns of *VvSS* genes were analyzed in the *V. vinifera* cv. Corvina global gene expression atlas, which consists of 54 different organs/tissues at various developmental stages obtained by microarray analysis (Supplementary Table S2). All *VvSS* genes had corresponding probes on the NimbleGen array. Figure 5A presents a graphical representation of the expression pattern of each *VvSS* gene. As a whole, the expression of five *VvSS* genes showed tissue- and organ-specific pattern (Figure 5A), in which *VvSS1*, *VsSS2*, and *VvSS5* showed a relatively low expression level, while *VvSS3* and *VvSS4* remained at higher expression levels in each tested tissue. Overall, *VvSS3* is highly expressed in vegetative tissues (berry pericarp, berry flesh, and berry skin) and transport tissues (rachis, tendril, and stem). However, except for expression overlap between rachis and tendril, *VvSS4* mainly presented higher transcript levels in bud, inflorescences, and seed, which are considered as reproductive organs. The expression pattern of these genes was also evaluated by qRT-PCR in selected organs of the *V. vinifera* grapevine cultivar (Figure 5B).

In order to complement the whole-transcriptome data, steady-state mRNA levels of all *VvSS* genes were investigated by real-time PCR in various organs/tissues of the *V. vinifera* grapevine cultivar, including root, tendril, stem, leaves, flower, berry and bud at different developmental stages. As shown in Figure 5B, grape *SS* transcripts were detected in a wide range of tissues and showed distinct but partially overlapping expression patterns, suggesting that *SS* genes may be implicated in a range of physiological processes in grapevine plant. Among these, *VvSS3* and *VvSS4* expression was detected in all tissues examined. However, *VvSS3* showed strong expression in all the tissues but *VvSS4* was found to be highly expressed in stem, flower, mid-ripening berry and burst bud, and relatively lower expression was noted in root, tendril, senescencing leaf, véraison berry and latent bud. While *VvSS1* was highly expressed in leaves and buds, and found that the expression of *VvSS1* peaked at the initial stage of leaf development (young leaves), and then decreased rapidly during leaf development to a low level at the mature stage. *VvSS2* was weakly expressed in the root and inflorescence. The very low expression levels of *VvSS5* were observed in tendril and ripening berry.

### 3.7. Expression Patterns of VvSS Genes in Response to Biotic and Abiotic Stresses

The expression of *VvSS* genes in response to both biotic and abiotic stresses was investigated using microarray data from several previously published papers. Regarding abiotic stresses, expression datasets were obtained from two studies (GSE31594 and GSE31677) conducted on transcriptomic response in leaves of *V. vinifera* cv “Cabernet Sauvignon” to short-term salt, water and cold stress, and long-term water and salt stress. Since the Affymetrix array used in these analysis was based on the few *VvSSs* sequences (cDNA and expressed sequence tags (ESTs)) known at the time, the expression of only a limited number of *VvSS* genes (*VvSS2*, *VvSS3* and *VvSS4*) could be determined (Supplementary Table S4).

**Figure 6:**
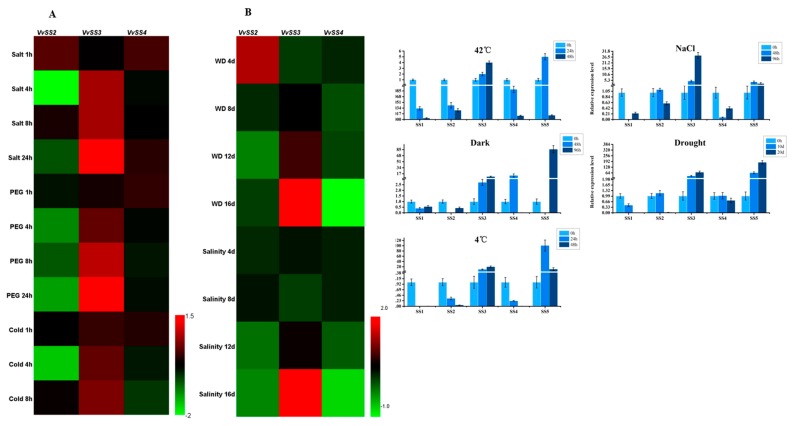
Expression profiles of *VvSS* genes in response to abiotic stresses. Expression of *VvSS* genes in the *V. vinifera* cv “Cabernet Sauvignon” (microarray) or two-year-old grapevines of *V. vinifera* cv. Chardonnay seedlings (qRT-PCR) in response to high temperature (42 °C), 100 mM NaCl, dark, drought (WS) and cold (4 °C). Microarray analysis presented in (**A**,**B**). qRT-PCR data presented in **C**. Microarray data were downloaded from the NCBI GEO datasets (GEO. Available online: https://www.ncbi.nlm.nih.gov/geo/) (GSE31594 and GSE31677), processed as log2 of the ratio between treated and untreated samples and graphically represented with HemI software. (**A**) *V. vinifera* cv “Cabernet Sauvignon” plants grown in a hydroponic drip system were treated with 120 mM NaCl, polyethylene glycol (PEG), cold (5 °C) or left untreated. Shoots with leaves were collected at 0, 1, 4 and 8 h for all treatments, and at 24 h for all treatments except cold (GEO series GSE31594). (**B**) Potted *V. vinifera* cv “Cabernet Sauvignon” vines in the greenhouse were exposed to a water-deficit stress (WD) by withholding water or a salinity stress by watering plants with a saline solution for 16 days. Non-stressed, normally watered plants served as the control for both treatments. Shoot tips were harvested every four days (0, 4, 8, 12 and 16 days) (GEO series GSE31677). (**C**) qRT-PCR expression analysis of *VvSSs* in *V. vinifera* cv. Chardonnay seedlings subjected to stress treatments. Transcripts were normalized to the actin gene expression. The mean ± s.d. of three biological replicates are presented.

In general, *VvSS2* was down-regulated in all stress treatments, and the expression of *VvSS4* was unaffected in some cases (salt, polyethylene glycol (PEG) and cold) and down-regulated in others (water deficit and salinity), while *VvSS3* gene showed significant changes in the patterns of expression levels. We found that *VvSS3* underwent continuous and significant increase after stress treatment (Figure 6). The data presented confirmed the putative involvement of *VvSS3* in abiotic stresses responses.

To further determine whether the *SS* genes were involved in abiotic stress resistance and validate the results obtained from the analysis of the microarray data, we measured their transcript levels under drought, salt, dark, and high and low temperature using qRT-PCR. As shown in Figure 6, *VvSS3* and *VvSS5* transcript abundance increased in all five treatments, *VvSS1* showed continuously low transcript levels in all treatments. When suffering high temperature stress, the expression levels of all of the *VvSS* genes were down-regulated, except for *VvSS3* and *VvSS5*, for which the transcript levels were up-regulated significantly. *VvSS5*, the most rapidly responding gene, reached a peak of nearly five-fold at 24 h post treatment (hpt) and rapidly decreased at 48 hpt. We also measured the transcript levels of *VvSS* genes in response to low temperature (4 °C) stress. Although transcript levels varied among the family members, *VvSS* genes exhibited a similar expression pattern among high and low temperature stress. Similarly, *VvSS5* responded strongly with transcript levels increasing to 90.0-fold at 24 h cold stress and then decreased rapidly around 15-fold at 48 h treatment.

After NaCl treatment, only *VvSS2*, *VvSS3*, and *VvSS5* showed up-regulation, although the expression levels of *VvSS2* and *VvSS5* were merely slightly increased (less than twofold) under this treatment. *VvSS3* was first declined and then rapidly up-regulated, reached a peak of 26.5-fold at 96 hpt, For the dark treatment, only *VvSS3*, *VvSS4*, and *VvSS5* showed up-regulation. *VvSS3* and *VvSS4* had a peak of 2.5-, and 15.5-fold respectively at 48 hpt but then their transcript levels rapidly decreased at the 96 h. We found *VvSS5* significantly responded to dark treatment (the peak is over than 70.0-fold at 96 hpt). As for the drought treatment, *VvSS1* and *VvSS4* shared similar expression patterns, with a slow increase in transcript levels to a peak and rapidly decrease. *VvSS3* and *VvSS5* also shared similar expression patterns, with a significantly increase in transcript levels to a peak after 20-day drought treatment.

Taken together, our results showed that *VvSS* genes expression varies in response to different abiotic stresses, indicating diverse roles of the *VvSS* genes in response to abiotic stress.

In regards to *VvSS* expression in response to biotic stresses, expression datasets obtained from three different host-pathogen interaction experiments were examined, including: the inoculation of *Erysiphe necator* on leaves of the susceptible *V. vinifera* cv. Cabernet sauvignon and the tolerant *V. aestivalis* cv. Norton [46] (Supplementary Table S4); infection of *V. Vinifera* cv. Chardonnay and cv. Incrocio Manzoni infection with the *Bois Noir* phytoplasma [47] (Supplementary Table S4); and the infection of *V. vinifera* cv. Cabernet Sauvignon with grapevine leaf-roll-associated virus-3 (GLRaV-3) during véraison and ripening stages of berry development [48] (Supplementary Table S4).

**Figure 7:**
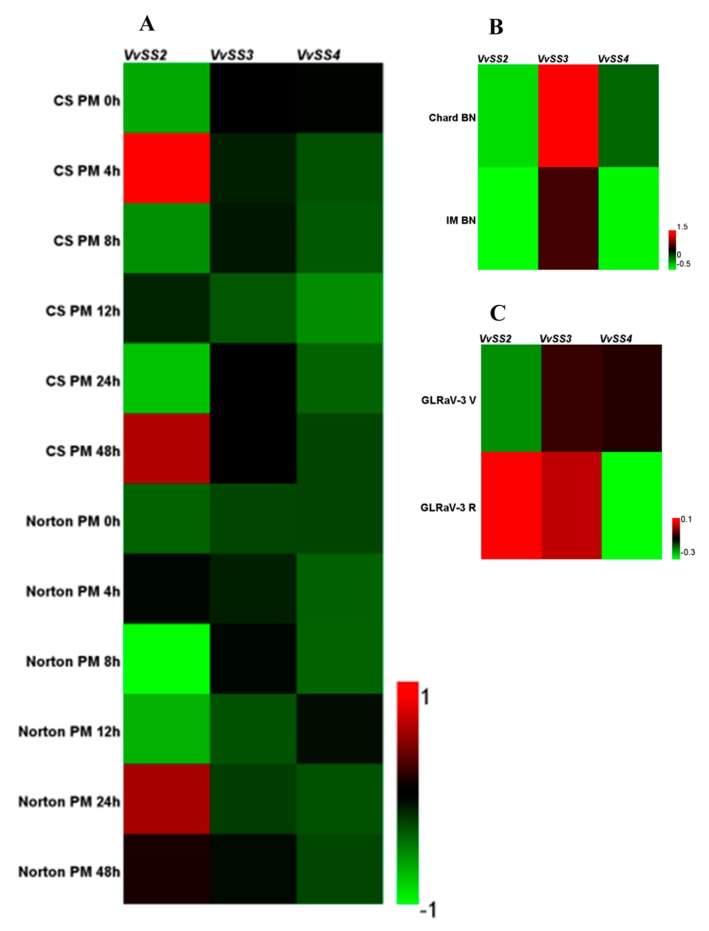
Expression profiles of *VvSS* genes in response to biotic stresses. (**A**) *V. vinifera* cv “Cabernet sauvignon” (CS) and *Vitis aestivalis* cv “Norton” plants were grown in an environmental chamber and inoculated with *Erysiphe necator* conidiospores (PM). Inoculated leaves were harvested at 0, 4, 8, 12, 24 and 48 h after inoculation (GEO series GSE6404). (**B**) Field-grown plants of *V. vinifera* cv “Chardonnay” (Chard) and “Incrocio Manzoni” (IM) naturally infected with *Bois Noir* phytoplasma (BN), compared to healthy samples (GEO series GSE12842). (**C**) *V. vinifera* cv “Cabernet Sauvignon” was infected with GLRaV-3 during véraison (V) and ripening (R) stages of berry development (GEO series GSE31660).

The effect *E. necator* infection on *VvSS* response appeared to be much stronger in the susceptible cv. Cabernet sauvignon than in the resistant cv. Norton (Figure 7A). *VvSS2* gene was upregulated in Cabernet sauvignon after 4 h of inoculation and in Norton it was upregulated after 24 h of inoculation.

Phytoplasma infection led to an induction of *VvSS3* genes in susceptible *V. Vinifera* cv. Chardonnay compared to the tolerant cv. Incrocio Manzoni (Figure 7B), and a repression of *VvSS2* and *VvSS4* (Figure 7B). Finally, the expression profiles of *VvSS* genes in response of grapevine to GLRaV-3 infection in vèraison phases revealed a general repression of the *VvSS* family (Figure 7C). However, a significant-upregulation, limited to *VvSS2* and *VvSS3*, specifically induced during the ripening phase.

## 4. Discussion

Comparative genomics approaches have been used to analyze *SS* gene families in various plant species, including citrus, tobacco, poplar, and cotton [28,29,49,50]. In the present study, we verified five *SS* genes from grape (*V. Vinifera*). These grape *SS* family members shared two conserved domain; we named these as *VvSS1–5*, according to distinct molecular characteristics. Given the limited knowledge of the grape *SS* gene family prior to this study, Comprehensive understanding of the evolutionary relationships, molecular structures, and expression profiles provides the important resources for their molecular mechanisms and possible functions in grape growth and development.

### 4.1. Evolutionary Conservation and Divergence Among Grape SS Genes

In all plant species examined to date, the SS isoenzymes are encoded by a small, multi-gene family [8,29,40,50]. Comprehensive analysis of this multi-gene family, including its exon/intron structures, phylogeny, and syntenic analysis, makes it possible for researchers to predict the potential functions and evolutionary relationships among uncharacterized members of this gene family.

Since gene exon/intron structures are typically conserved among homologous genes of a gene family [51], analysis of exon/intron structures can provide a clue to reveal the evolutionary history of certain gene family [52]. Previous detailed studies of the *SS* genes shed light on highly conservation in the length, number and position of exon/introns in several distantly related dicot and monocot plants, bring about the speculation that the divergence of the three ancestors prior to the segregation of monocot and eudicot species [53]. In the present study, the predicted molecular features of the grape SS proteins were similar to those of previously characterized SS proteins from other plant species. We also estimated the exon/intron structures of the grape *SS* genes (Figure 2, Figure S2). The integrated gene structure model in *VvSS* was very similar to that of *SS* genes from tobacco [50], poplar [29], cotton [28] and Arabidopsis and rice [27,31]. For instance, the position of exons of five putative *SS* genes showed parallel positions, and most of the exons between Group I, II and III shared a high level of similarity but only existed difference at two ends of sequence. However, genome-wide duplication events and subsequent chromosomal rearrangements have differentially shaped the *SS* family of grape. For example, the numbers and length of introns of five *VvSS* genes were different greatly (Figure 2 and Figure S2), whereas the number and positions of exons were highly conserved among *SS* genes in citrus, tobacco, poplar, and cotton [28,29,49,50]. In addition, the arrangement and length of intron/exon of *VvSS2* and *VvSS3* showed the antiparallel relationship. We further analyzed whether these phylogenetic groups corresponded to the *VvSS* genes structure models. Indeed, clustering the intron/exon organizations of the five *SS* genes by an unrooted phylogenetic tree suggests a connection between intron/exon structures and evolutionary history.

The conservation and divergence of grape *SS* genes’ structure led to the expansion of gene family members and functional conservation/differentiation of gene. In general, three pivotal mechanisms contribute to gene family evolution and expansion: exon/intron gain or loss, exonization/pseudo-exonization, and insertion/deletion [17]. For the three groups in Figure 2, according to the phylogenetic relationships of five *VvSS* genes in the present study (see the contents below) and previous work in other plants [49,50], Group III (*VvSS1/VvSS5*) was the earliest one that expanded from the evolutionary branch. As a result, *VvSS1/VvSS5* has the longest evolutionary history, leading to the complex of intron/exon structure. All members of Group I and II lack two exons and two introns at the last position, indicating that there was an intron deletion near the last exon, which increased the number of the *VvSS* family members. The divergence of intron/exon was closely related to the evolutionary history of the grape *SS* family and might result in functional diversification.

To further study the molecular evolution and explore their possible function of the *VvSS* gene family, we also investigated gene duplication events, and syntenic and phylogenetic relationships of the *VvSS* genes. Segmental duplications and tandem duplications can lead to increasing or decreasing copy numbers in gene families [54]. These processes may lead to functional redundancy, sub-functionalization and neo-functionalization. A segmental duplicated pair (*VvSS2–VvSS3)* in the grapevine genome was found, but no tandem duplicated *VvSS* genes were discovered (Figure 3, Supplementary Table S1). The duplicated gene pair (*VvSS2–VvSS3*) had high similarities in gene length, protein properties, and intron/exon structure (Tables S1 and S2, Figure 2). Furthermore, the gene pair (*VvSS2–VvSS3*) showed the tight phylogenetic relationship among plant *SS* gene families and within grape *SS* genes (Figure 2 and Figure 4). They also showed closer phylogenetic relationships with soybean *SS* genes than that with each other, and these two genes have different syntenic genes in soybean (*G. max*) (Figure 3, Supplementary Table S1), indicating that the duplication events happened before the divergence of the grape and soybean lineages. As discussed above, *VvSS2* and *VvSS3* likely have functional redundancy.

Comparative genomics relies on the structuring of genomes into syntenic blocks that exhibit conserved features across the genomes [55]. The syntenic analysis provides evolutionary and functional characterization of *SS* gene families in grape and soybean. In this work, all five grape *SS* genes were found to have syntenic relationships with soybean genes (Figure 3). Thus, a large number of syntenic relationships indicated that some of the grape *SS* genes arose before the divergence of these two species. According to the phylogenetic tree, *VvSS* genes established closer phylogenetic relations with the corresponding *GmSS* genes (Figure 4), suggesting strongly some level of functional similarities.

Previous studies of the molecular structures and phylogenetic relationships of plant *SS* gene families divided *SS* genes into three groups (SS1, SSA, and New Group) [26,27]. This classification was corroborated a number of subsequent studies, and three groups were respectively named as the Class I, Class II, and Class III groups [2,28,56]. Later, the Class I group can be further divided into a monocot subgroup and a eudicot subgroup, as these two subgroups were obviously distinct from each other in phylogenetic trees constructed by dozens of plant *SS* gene families. The Class II and Class III groups were then classified into mix group 1 and mix group 2, as members of these groups contained monocot and eudicot plants [29]. In our study, we performed phylogenetic analysis of the *SS* genes from grape and fifteen other plant species, and found that the grape *SS* genes family had at least one member in three single groups: *VvSS4* in Class I, *VvSS2* and *VvSS3* in Class II and *VvSS1* and *VvSS5* in Class III. The five *VvSS* genes in the dicot group were divided into three subgroups, specifically, *VvSS1* and *VvSS5* clustered closely together, *VvSS2* and *VvSS3* clustered together, while *VvSS4* alone clustered into one branch. *VvSS1* and *VvSS5* were apparently apart from Arabidopsis *SS* genes. This result demonstrated that the gene duplication event that generated *VvSS1* and *VvSS5* gene occurred after monocot–eudicot separation, but before the divergence of *Vitaceae*/*Arabidopsis*. In addition, the generation of the *VvSS2* and *VvSS3* genes probably took place after the separation of *Vitaceae*/*Arabidopsis*. Grape, sweet orange, and apple are both members of Rosidae, possessed a close evolutionary relationship. There were no sweet orange and apple *SS* genes show closely relationship with *VvSS4*, suggesting that *VvSS4* should be more recent than the differentiation between grape and sweet orange, apple. Thus, *VvSS1* and *VvSS5* were older than the other grape *SS* genes, the diverged duplications of *VvSS4* have occurred more recently and may split after the divergence of the Rosidae, and grape might have lost a copy after its divergence from Arabidopsis compared with the gene copy number of Arabidopsis. We also found positive selection contributed to evolution of this gene family. Furthermore, Tajima relative rate tests identified accelerated evolutionary rates in *VvSS2/VvSS3* duplicates (Table 4). Based on the genome of *V. vinifera* [42], and the facts that grape *SS* family was distributed on five chromosomes (Figure 2), our phylogenetic analysis, the exon/intron structure, and *VvSS2* and *VvSS3* located in systemic blocks, we propose an interpretation of evolutionary history of the grape *SS* genes. Before the monocot–eudicot divergence, an early gene duplication in the ancestor gave rise to the three progenitors of the three *SS* groups with conserved exon/intron structure. Two of the three precursors evolved independently and finally retained one single gene in the Class II group (*SS2*) and Class III group (*SS1*), respectively. After the divergence of monocots and eudicots, duplication (may be whole-genome duplication event) of the *SS* precursor generated *SS3* and *SS5*, whereas *SS4* were produced after differentiation within the Rosidae (Figure 4).

### 4.2. VvSS Genes Involved in Grapevine Growth and Development

Functional diversity caused by the gene duplication resulted in the altered expression profiles and/or protein property, and it was a major evolutionary driver to increase the fitness to new environment of plants [57]. Analysis of gene expression profiles can be used in some level to predict the physiological processes genes involved in. To date, although the detailed expression profiles of *SS* genes have been examined in other plant species, such as Arabidopsis, rice, cotton, poplar, and rubber tree [2,22,25,27,28,29], there have been no detailed surveys of the expression of grape *SS* genes.

In the present study, the expression patterns of *VvSS* genes in different grapevine tissues and at different developmental stages were examined using an expression atlas of *V. vinifera* cv Corvina. The analysis revealed that the abundant expression of *VvSS3* and *VvSS4* gene in specific grapevine tissues, possibly reflected their involvement in a common metabolic and/or developmental process (Figure 5).

The expression analysis revealed that *VvSS3* were highly expressed in vegetative and transport tissues, suggesting a key role of *VvSS3* in the regulation of sugar accumulation in berry and sugar transport in stem and tendril of grapevine. *VvSS4* was found to be specifically expressed in reproductive tissues and highly accumulated during flower development, which was shown to may have a role in energy supplying to support the process from bud to flower. Similarity, the spatial-temporal expression of *SS* genes has been previously reported in many other plants. For example, at the early stage of fruit development of apple, the transcript levels of *SS* are high. As the fruit continues to grow due to cell expansion, the transcript levels of *SS* were down-regulated [58]. For tissues and organs, *CitSus1* and *CitSus2* were predominantly expressed in fruit juice sacs (JS), whereas *CitSus3* and *CitSus4* were predominantly expressed in early leaves (immature leaves), and *CitSus5* and *CitSus6* were predominantly expressed in fruit JS and in mature leaves. During fruit development, *CitSus5* transcript increased significantly and *CitSus6* transcript decreased significantly in fruit JS. In addition, in tobacco, *Sus2* (Ntab0259170) and *Sus3* (Ntab0259180) gene based on high transcript levels also play a predominant role in sucrose metabolism during leaf development [49]. Zou et al., (2013) [28] demonstrated that most *Sus* genes were differentially expressed in various tissues and *GrSUS1*, *GrSUS3*, and *GrSUS5* showed significantly higher expression levels and underwent significant changes in expression during fiber development in the three *Gossypium* species.

*VvSS1*, *VvSS2* and *VvSS5* genes were not expressed, or were expressed at low levels, suggesting their redundant function in the normal development and growth process of grape. However, in certain tissues, other *VvSS* genes except for *VvSS3* and 4, also plays an important role. For example, *VvSS2* was barely expressed at a high level in seed-PFS, and *VvSS1* was similarly expressed at a high level in tendril, and tendril. These results suggest that the functions of *VvSS* genes are diversified and yet partially overlap. Since sucrose affects cell division and vascular tissue differentiation in plant leaves, *VvSS4* remained at a steady high level, and *VvSS3* significantly decreased during the course of leaf development (which is same expression pattern in bud). Overall, comparison of the transcripts of all *VvSS* genes in each single tissue revealed the predominant role of *VvSS3* and *VvSS4* isoforms in the growth and development of grapevine.

### 4.3. Stress Induced VvSS Expression in Grapevine

Sucrose synthases have been reported to be associated with plant responds to various environmental stresses. For instance, in Arabidopsis, expression of the *AtSS1* gene could be induced by cold or drought treatment. *AtSS3* is used as a molecular marker of dehydration [25]. In addition, two barley *SS* genes (*HvSS1* and *HvSS3*) and one rubber tree *SS* gene (*HbSS5*) significantly responded to low temperature and drought stresses [22,59]. The higher expression levels of *SS* genes may result from the increased glycolytic demand under abiotic stresses [60].

In our study, the transcriptomic databases generated in previous studies of grapevine subjected to biotic and abiotic stresses, together with our qRT-PCR analysis, allowed us to identify *VvSS* genes putatively involved in stress response.

The members of grape *SS* gene family exhibited different responses of expression patterns in the mature leaf in response to different abiotic stress. In detail, under all abiotic stress, the transcription levels of *VvSS3* and *VvSS5* were continuously up-regulated, and thus the key enzymes of *VvSS3* and *VvSS5* encoded might be the predominant isoforms of leaf to respond to abiotic stress. Simultaneously, the transcription levels of the other *VvSS* genes were significantly down-regulated after abiotic stress treatment. The similar expression response of *SS* genes was also found in rubber tree (*Hevea brasiliensis*) [22] and barley (*Hordeum vulgare*) [59]. Low temperature and drought treatments conspicuously induced *HbSus5* expression in root and leaf, suggesting a role in stress responses of rubber tree. Only *HvSs1* is up-regulated by anoxia and cold temperatures from the four *SS* genes in barley. In addition, expression of *Sus5* (*Ntab0288750*) and Sus7 (*Ntab0234340*) were conspicuously induced by low temperature and virus treatment, indicating that these two isozymes are important in meeting the increased glycolytic demand that occurs during abiotic stress [50].

Regarding biotic stresses, to date the involvement of *VvSS* genes in biotic responses has been not previously reported. In this study, a significant induction of *VvSSs* did not occur in response to *E. necator*, *Bois Noir*, and GLRaV-3 infection. In contrast, a significant repression of *VvSSs* was observed, suggesting that these *VvSS* genes may be not involved in the pathogen response pathway. It is important to note that despite showing a high fold change of *VvSS2* in infected vs. mock-inoculated leaves in the biotic stresses, the baseline levels of *VvSS2* transcript were always lower (Supplementary Table S1). However, *VvSS3* was significantly upregulated in response to *Bois Noir* phytoplasma infection, indicating that *VvSS3* might play an important role in plant defense against *Bois Noir*.

In addition, the duplicated gene pair, *VvSS2*–*VvSS3* possessed apparently unique expression profiles, further suggesting that the duplicated gene pair *VvSS2*–*VvSS3* might have undergone sub-functionalization. It was inconsistent with the previous research that members of the same *SS* genes orthologous group had very similar expression patterns in all three *Gossypium* species [28].

Overall, members of the grape sucrose synthase family exhibited different expression patterns in different tissues and in response to biotic and abiotic stresses. Despite many recent advances in functional studies of *SS*s in grapevine, the biological function of most *VvSS* genes in physiological and developmental processes and plant defense still needs to be elucidated. The bioinformatic analysis and expression patterns of the *VvSS* gene family conducted in the present study provide an overall picture of the composition and expression of *sucrose synthase* genes in grapevine that will facilitate selecting candidate genes for cloning and further functional characterization.

## 5. Conclusions

Five *sucrose synthase* genes in grapevine were bioinformatically identified and characterized. We propose that *VvSS1/VvSS5*, *VvSS2/VvSS3* and *VvSS4* originated from three ancient *SS* genes, respectively, which were generated by duplication events before the split of monocots and eudicots. The spatio-temporal expression profiles of the *VvSS* genes in various tissues at various developmental stages suggested *VvSS3* and *VvSS4* play a predominant role in sucrose metabolism during the growth and development grape. Expression of *VvSS3* was significantly induced by stress treatment, indicating that the isozyme played an important role in releasing stress.

## Figures and Tables

**Table 1:** Characteristics of Grape *Sucrose Synthase* (*SS*) genes.

Name	Locus Id ^a^	Genomic DNA Size (bp)	CDS Size (bp)	Number of Amino Acids	Predicted Mw (kDa)	Theoretical pI	Ai	GRAVY	Chromosome Location	Group	Functional Domains (Start–End, bp)	
											Sucrose Synthase	Glycosyl Transferase
*VvSS1*	VIT_204s0079g00230	4513	2457	840	95.4	8.28	83.49	−0.339	chr4:10519070-10523582	II	9-557	569-745
*VvSS2*	VIT_205s0077g01930	7914	2508	835	95.6	5.85	91.99	−0.244	chr5:1507610-1515524	I	8-553	566-765
*VvSS3*	VIT_207s0005g00750	9214	2436	811	92.4	5.73	90.49	−0.254	chr7:3380739-3389952	I	8-555	568-741
*VvSS4*	VIT_211s0016g00470	4229	2448	815	93.6	6.13	92.20	−0.247	chr11:489955-494383	I	9-553	565--740
*VvSS5*	VIT_217s0053g00700	4820	2610	906	102.7	7.55	81.19	−0.359	chr17:15994677-15999496	II	12-558	568-736

^a^ IDs are available in the CRIBIV2.1 database. Abbreviations: Ai, aliphatic index; GRAVY, grand average of hydropathicity; MW, molecular weight; pI, isoelectric point; CDS, Coding DNA sequence; Chr, chromosome numbers.

**Table 2:** Coding region nucleotide (upper portion of matrix) and amino acid (bottom portion of matrix) sequence pairwise comparisons (% similarity) between grape sucrose synthase genes.

	*VvSS1*	*VvSS2*	*VvSS3*	*VvSS4*	*VvSS5*
*VvSS1*	-	51.79	53.81	53.45	70.56
*VvSS2*	59.18	-	79.12	66.86	47.70
*VvSS3*	59.93	77.55	-	71.15	50.00
*VvSS4*	59.60	66.36	69.47	-	59.57
*VvSS5*	70.50	54.85	56.60	56.85	-

**Table 3:** Divergence between paralogous *SS* gene pairs in grape.

Gene 1	Gene 2	dN	dS	dN/dS
*VvSS2*	*VvSS3*	0.3012	0.0957	3.1473
*VvSS1*	*VvSS5*	0.2759	0.1246	2.2143

dN: nonsynonymous site; dS: synonymous site.

**Table 4:** Tajima relative rate tests of *SS* gene pairs in grape ^a^.

Testing Group	Mt ^b^	M1 ^c^	M2 ^d^	X^2^	*P* ^e^
*VvSS2/VvSS3* with *GmSS2*	586	11	30	8.91	0.00450
*VvSS1/VvSS5* with *GmSS4*	472	9	4	1.92	0.21690

^a^ The Tajima relative rate test was used to examine the equality of evolutionary rate between grape gene pairs; ^b^ Mt is the sum of the identical sites and the divergent sites in all three sequences tested; ^c^ M1 is the number of unique differences in the first paralog; ^d^ M2 is the number of unique differences in the second paralog; ^e^ If *P* < 0.05, the test rejects the equal substitution rates between the two duplicates and infers that one of the two duplicates has an accelerated evolutionary rate.

## Supplementary Materials

The following supplementary material can be found online at www.mdpi.com/2073-4425/8/4/111/s1, Table S1 Syntenic relationships among grape and soybean sucrose synthase genes and within grape *SS* genes. Table S2 List of sucrose synthase gene sequences used in this study. Table S3 Gene-specific primers used for quantitative RT-PCR analysis of *VvSS* gene expression. Table S4 Microarray data of *VvSS* genes downloaded from the GEO datasets.
