# Supplementary material for: Genome-Wide Analysis of the Sucrose Synthase Gene Family in Grape (Vitis vinifera): Structure, Evolution, and Expression Profiles
Source: Genes (Basel). 2017 Mar 28;8(4):111. doi: 10.3390/genes8040111 (PMC5406858; doi:10.3390/genes8040111)
Supplement: Supplementary file 1 [file genes-08-00111-s001.zip › Table S1.docx]

| Block No | Block Score | E Value | Locus 1 | Locus 2 | Ka | Ks |
| --- | --- | --- | --- | --- | --- | --- |
| 127 | 637 | 0 | Glyma.02G240400 (*GmSS1*) | VIT_204s0079g00230 (*VvSS1*) | 0.1421 | 1.3173 |
| 721 | 214 | 0 | Glyma.09G167000 (*GmSS4*) | VIT_204s0079g00230 (*VvSS1*) | 0.1768 | 1.9164 |
| 1145 | 679 | 0 | Glyma.14G209900 (*GmSS6*) | VIT_204s0079g00230 (*VvSS1*) | 0.1384 | 1.3406 |
| 200 | 525 | 0 | Glyma.03G216300 (*GmSS2*) | VIT_205s0077g01930 (*VvSS2*) | 0.1196 | 1.2452 |
| 1216 | 622 | 0 | Glyma.15G151000 (*GmSS8*) | VIT_205s0077g01930 (*VvSS2*) | 0.1063 | 0.8842 |
| 1484 | 565 | 0 | Glyma.19G212800 (*GmSS11*) | VIT_205s0077g01930 (*VvSS2*) | 0.1182 | 1.2471 |
| 209 | 3768 | 0 | Glyma.03G216300 (*GmSS2*) | VIT_207s0005g00750 (*VvSS3*) | 0.0987 | 1.1127 |
| 1491 | 3932 | 0 | Glyma.19G212800 (*GmSS11*) | VIT_207s0005g00750 (*VvSS3*) | 0.0964 | 1.0578 |
| 680 | 2574 | 0 | Glyma.09G073600 (*GmSS3*) | VIT_211s0016g00470 (*VvSS4*) | 0.119 | 1.3423 |
| 1016 | 3317 | 0 | Glyma.13G114000 (*GmSS5*) | VIT_211s0016g00470 (*VvSS4*) | 0.0989 | 1.1884 |
| 1182 | 2538 | 0 | Glyma.15G182600 (*GmSS7*) | VIT_211s0016g00470 (*VvSS4*) | 0.1085 | 1.273 |
| 1292 | 3400 | 0 | Glyma.17G045800 (*GmSS10*) | VIT_211s0016g00470 (*VvSS4*) | 0.1013 | 1.284 |
| 708 | 575 | 0 | Glyma.09G167000 (*GmSS4*) | VIT_217s0053g00700 (*VvSS5*) | 0.2015 | 1.3794 |
| 1261 | 610 | 0 | Glyma.16G217200 (*GmSS9*) | VIT_217s0053g00700 (*VvSS5*) | 0.202 | 1.3373 |
| 119 | 1128 | 0 | GSVIVT01028043001 (*VvSS3*) | VIT_217s0053g00700 (*VvSS2*) | 0.1072 | 0.9003 |
