# Supplementary material for: Genome-Wide Analysis of the Sucrose Synthase Gene Family in Grape (Vitis vinifera): Structure, Evolution, and Expression Profiles
Source: Genes (Basel). 2017 Mar 28;8(4):111. doi: 10.3390/genes8040111 (PMC5406858; doi:10.3390/genes8040111)
Supplement: Supplementary file 1 [file genes-08-00111-s001.zip › Table S3.doc]

| Gene | Forward primer (5′-3′) | Reverse primer (5′-3′) |
| --- | --- | --- |
| *VvSS1* | ACATGGAGAGACACAAGCGA | AACCACTCAGTTAACCCCGT |
| *VvSS2* | GACTACTGACACTGGCTGGG | AGTGGAATGGAAGTCGCCAG |
| *VvSS3* | GCCCTGCATGGTTCAATTGA | GTCAAGCCTTGCCATGGAAA |
| *VvSS4* | TGGCTACTTTGCTCAGGACA | GTCCTTGCTGCTTGATACGG |
| *VvSS5* | AGCCCGTCCTCAACACTAAA | TGTGGGCAAGGATCTTAGCA |
